# Transient Oligomerization of the SARS-CoV N Protein – Implication for Virus Ribonucleoprotein Packaging

**DOI:** 10.1371/journal.pone.0065045

**Published:** 2013-05-23

**Authors:** Chung-ke Chang, Chia-Min Michael Chen, Ming-hui Chiang, Yen-lan Hsu, Tai-huang Huang

**Affiliations:** Institute of Biomedical Sciences, Academia Sinica, Taipei, Taiwan, Republic of China; 2 Department of Physics, National Taiwan Normal University, Taipei, Taiwan, Republic of China; Centro de Biología Molecular Severo Ochoa (CSIC-UAM), Spain

## Abstract

The nucleocapsid (N) phosphoprotein of the severe acute respiratory syndrome coronavirus (SARS-CoV) packages the viral genome into a helical ribonucleocapsid and plays a fundamental role during viral self-assembly. The N protein consists of two structural domains interspersed between intrinsically disordered regions and dimerizes through the C-terminal structural domain (CTD). A key activity of the protein is the ability to oligomerize during capsid formation by utilizing the dimer as a building block, but the structural and mechanistic bases of this activity are not well understood. By disulfide trapping technique we measured the amount of transient oligomers of N protein mutants with strategically located cysteine residues and showed that CTD acts as a primary transient oligomerization domain in solution. The data is consistent with the helical oligomer packing model of N protein observed in crystal. A systematic study of the oligomerization behavior revealed that altering the intermolecular electrostatic repulsion through changes in solution salt concentration or phosphorylation-mimicking mutations affects oligomerization propensity. We propose a biophysical mechanism where electrostatic repulsion acts as a switch to regulate N protein oligomerization.

## Introduction

A fundamental part of viral self-assembly is the packaging of the viral genome into ribonucleoprotein (RNP) complexes called capsids. Capsid formation requires two key activities: the interaction between protein and nucleic acid and the ability of the complex to oligomerize [1]. The severe acute respiratory syndrome coronavirus (SARS-CoV) nucleocapsid (N) protein is a phosphoprotein that performs both functions [2]. While the interaction between SARS-CoV N protein and nucleic acids has been examined at the genetic, biochemical and biophysical levels, understanding of SARS-CoV N protein oligomerization is relatively limited. Although it is generally accepted that the N protein dimer serves as the basic building block of the nucleocapsid in coronaviruses, the structural and mechanistic bases of how N protein dimers oligomerize to form larger entities remain a mystery [3], [4], [5], [6], [7], [8], [9].

**Figure 1:**
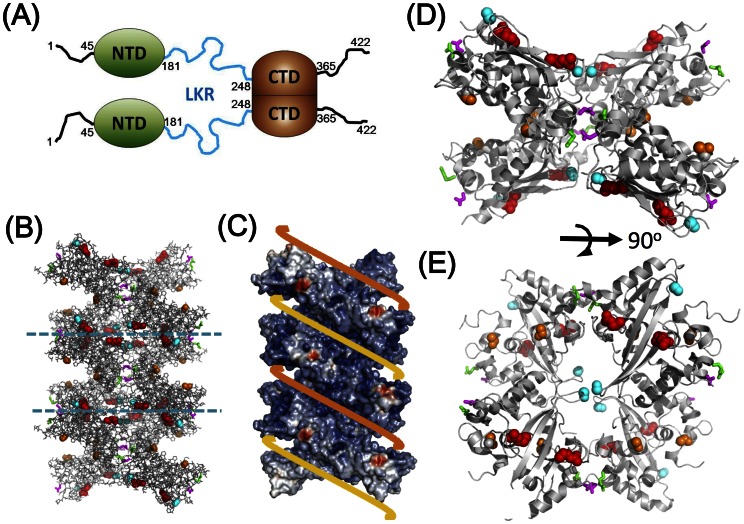
The structure packaging of the SARS-CoV N protein and the mutation sites for probing transient molecular interaction. (A) The domain organization of the N protein. (B) Side view of the crystal structure of 24-mer CTD molecules showing the helical packing. (C) Electrostatic surface of the crystal packing of the CTD 24-mer. Positive charges are colored in blue and negative charges are colored in red. Yellow and orange ribbons represent viral RNA strands wrapping around the helical oligomer structure. (D) A close up side view of a CTD octamer molecule shown in between of the two dashed lines on Fig. 1B. The corresponding top view is shown on (E). Spatial locations of the mutated sites are shown in orange (T264), magenta (Q290), green (R294), red (R320) or cyan (S328) in (B), (D) and (E).

SARS-CoV N protein consists of two structural domains interspersed between intrinsically disordered regions (IDR), a unique arrangement shared among coronaviruses [10] (Fig. 1A). The N-terminal structural domain (NTD, res. 45–181) adopts an OB (oligonucleotide binding)-like fold capable of binding to nucleic acids, whereas the C-terminal structural domain (CTD, res. 248–365) is responsible for dimer formation through a domain-swapping mechanism [3], [8], [9], [11]. We have shown that the CTD dimer is also capable of binding directly to nucleic acids [9], [12]. Intriguingly, IDRs also seem to modulate nucleic acid binding of SARS-CoV N protein, with at least one IDR, the flexible linker region (LKR, res. 182–247), capable of direct interaction with RNA under *in vitro* conditions [13]. Like many capsid proteins, SARS-CoV N protein has a high isoelectric point (pI), with a large excess of positively charged residues distributed throughout the protein (+6 charges between residues 176–204, the SR-rich region; +7 charges between residues 249–267 in CTD region; +8 charges between residues 370–389 in the C-terminal IDR region) that are important for the nucleic acid binding activity [11], [12], [13].

In the absence of nucleic acids, full-length SARS-CoV N protein predominantly exists as a dimer in solution [14]. However, a truncated mutant that includes part of the C-terminal structural domain and the C-terminal IDR (res. 283–422) was shown capable of forming high-order oligomers in a concentration-dependent manner [15]. X-ray crystallography results of the NTD and CTD imply that the two structural domains also have oligomerization potential, even though they showed no indication of forming oligomers in solution [16]. In particular, we have shown that the CTD packed in a helical conformation with dimension and charge distribution that conforms to that observed in EM structures (Figure 1B, 1C) [9], [17]. These studies suggest that SARS-CoV N protein could potentially form high-order oligomers by itself if protein concentrations were high enough, and small populations of oligomers could exist even at low concentrations, but could not be detected through conventional methods. Closer inspection revealed that the interface area between the dimers in the helical packed crystal structure of CTD is small, consistent with the inability to detect higher order CTD oligomer in solution. Nonetheless, such an oligomerization behavior may be relevant to RNP packaging.

SARS-CoV N protein is highly phosphorylated in cells, and tentative phosphorylation sites have been mapped to the Ser/Arg-rich portion of the LKR [18], [19], [20]. Notably, it has been suggested that the LKR is directly involved in N protein oligomerization [21]. A conflicting report, however, indicates that the LKR interfered with N protein oligomerization when the C-terminal domain is present [22]. Peng *et al* demonstrated that phosphorylation of the LKR by the SR protein kinase-1 (SRPK1) partially impaired the self-association of the full-length protein [19]. Although there is a connection between phosphorylation and N protein oligomerization, whether this is due to a simple charge effect, conformational changes or involves interactions with other components of the virus-host system remains unclear.

In this study, we used *in vitro* disulfide trapping and NMR spectroscopy to detect oligomeric species of the SARS-CoV N protein CTD in solution. We found that interactions between CTD dimers conform to that predicted from the helical crystal packing. Extending our findings to a di-domain construct (DD, res. 45–365) which includes the NTD, CTD and LKR, we conclude that the DD is also able to form oligomers through the CTD in solution. Introduction of negative charges to the LKR through phosphorylation-mimicking mutations in the DD construct resulted in enhanced oligomerization. We discuss the implications of our findings in the context of viral capsid assembly and propose a model where coronavirus N protein oligomerization is regulated by the modulation of charges on the protein.

## Materials and Methods

### Cloning, protein expression and purification

Various protein domains were cloned and expressed as described previously [8], [10]. For disulfide trapping experiments, we chose mutation sites that would form disulfide linkages based on the crystal packing structures of the SARS-CoV N protein CTD (Figure 1) [9]. Sites for the phosphomimicking mutations were chosen based on recent literature [18], [19], [20]. Standard PCR techniques were employed to obtain mutant clones, which were then confirmed through DNA sequencing. Mutant proteins were expressed in *Escherichia coli* BL21(*de3*) cells, followed by affinity and size-exclusion chromatography purification as previously described [8], [10]. Isotope-labeled proteins for NMR studies were prepared using established protocols [8], [10]. Purification of u-^15^N,^2^H-CTD_Q290C_ tetramer was performed by collecting the tetramer fraction from a Superdex 75/60 size exclusion column after treating the protein with 10 mM β-mercaptoethanol at 4°C for one hour and extensive washing with FPLC buffer (50 mM sodium phosphate, 150 mM NaCl, 1 mM EDTA, pH 7.4) in an Amicon centrifugal concentrator (Millipore, MA).

*NMR spectroscopy.* Samples contained 0.1–0.4 mM protein in NMR buffer containing 10 mM sodium phosphate, pH 6.0, 50 mM NaCl, 1 mM EDTA, 0.01% NaN_3_, 10% D_2_O and Complete Protease Inhibitor cocktail (Roche, Germany). Samples of Cys mutants also contained 10 mM dithiothreitol (DTT) to avoid formation of disulfide bonds. All experiments were performed on Bruker 600-MHz spectrometers equipped with cryoprobes at 30°C unless stated otherwise. The acquired data were processed with the TopSpin suite (Bruker Biospin, Germany) or iNMR (Nucleomatica, Italy).

### Disulfide trapping assay

Fixed amounts of purified proteins containing cysteine mutations (3 mg/ml for the CTD and 1.5 mg/ml for the DD) under various buffer conditions were allowed to react with 10 mM of β-mercaptoethanol for 1 hour at 4°C, followed by extensive washing on an Amicon centrifugal concentrator with FPLC buffer to remove the β-mercaptoethanol. Molecules in close contact and correct orientation spontaneously form disulfide linkages, which can be assayed through size exclusion chromatography and sodium dodecyl sulfate polyacrylamide gel electrophoresis (SDS-PAGE). Controls in the form of DD Q290C were performed for each experiment to ensure β-mercaptoethanol efficacy.

### Size exclusion chromatography

Experiments were conducted on an Akta Fast-Performance Liquid Chromatography (FPLC) System (GE Healthcare, CA) equipped with a Superdex 75/60 column at an elution rate of 1.0 ml/min. Apparent molecular weights of the proteins were estimated from the elution profile calibrated with the LMW Gel Filtration Calibration Kit (GE Healthcare, CA). Elution volume and molecular weight have the following relationship:
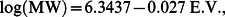
where MW is the molecular weight in Daltons and E.V. is the elution volume in ml. Peak quantification was achieved through analysis with Igor Pro software (WaveMetrics, OR), where each peak in the chromatogram was fit with a Gaussian curve and the underlying area integrated in the program. Area integration errors were estimated from the residual between fitted and experimental curves.

### Sodium dodecyl sulfate polyacrylamide gel electrophoresis

Disulfide trapping samples were boiled for 2 min at 94°C in the presence and absence of β-mercaptoethanol. The boiling time was kept short (2 min) to avoid protein aggregation. The samples were then loaded on a NuPage 4–12% Bis-Tris gel (Invitrogen, CA) and run at room temperature according to manufacturer's instructions. The gel was digitized on a UVP BioDoc-It equipped with a fluorescent plate (UVP, CA). Quantification of the disulfide-linked and unlinked bands was achieved through the ImageJ software (National Institutes of Health, MA).

### Structure images

The solution structure (PDB ID: 2JW8) and crystal structure (PDB ID: 2CJB) of the CTD spanning residues 248–365 were obtained from the Protein Data Bank (www.rcsb.org). All structure images were prepared with PyMOL (www.pymol.org).

## Results

### The CTD is a transient self-association site of the SARS-CoV N protein

Within the crystal asymmetric unit, the SARS-CoV N protein CTD packs as an octamer which stacks to form a helical arrangement with a continuous positively charged surface that could potentially allow the RNA to bind to it through electrostatic interactions (Fig. 1) [9]. Such organization suggests a RNA-binding facilitated mechanism for the formation of a helical RNP particle. It is unclear whether the oligomer structure is biologically relevant, since there have been no reports of oligomer species being detected in solution. To test the possibility that the oligomer structure reflects the existence of transient interactions that have been trapped during the crystallization process, we applied an *in vitro* disulfide trapping technique in an attempt to capture these transient interactions in solution. Since wild-type SARS-CoV N protein contains no cysteine residues, we engineered single-site cysteine mutations at various locations (T264C, Q290C, R294C, R320C, and S328C) on the CTD dimer. Q290 and R294 are located at helix 2 and S328 is located at the loop region connecting the two β-strands. Based on the crystal packing of the CTD structure as shown in Figures 1B-1E, both Q290 residues (colored magenta) in a CTD-dimer are located at the interface of the adjacent dimers in the oligomer. The distance between the β-carbons of the Q290-Q290 pair is within 8 Å, a suitable distance for disulfide trapping reactions (Figure 1C) [23]. Similarly, the β-carbons of the R294-R294 pair (colored green) are also within a distance suitable for disulfide bond formation. On the other hand, S328 residues (colored cyan) of the CTD dimer are located in the central cavity of the CTD super-helix and are in a favorable position to form inter-dimer disulfide bonds with another S328 residue. The rest of the mutations are not located at the interface and were engineered as negative controls. The mutants did not perturb the structure of the CTD when monitored through ^15^N-edited heteronuclear single-quantum coherence (HSQC) NMR spectroscopy as judged by the absence of chemical shift perturbation greater than 0.03 ppm other than that of the mutated residues.

**Figure 2:**
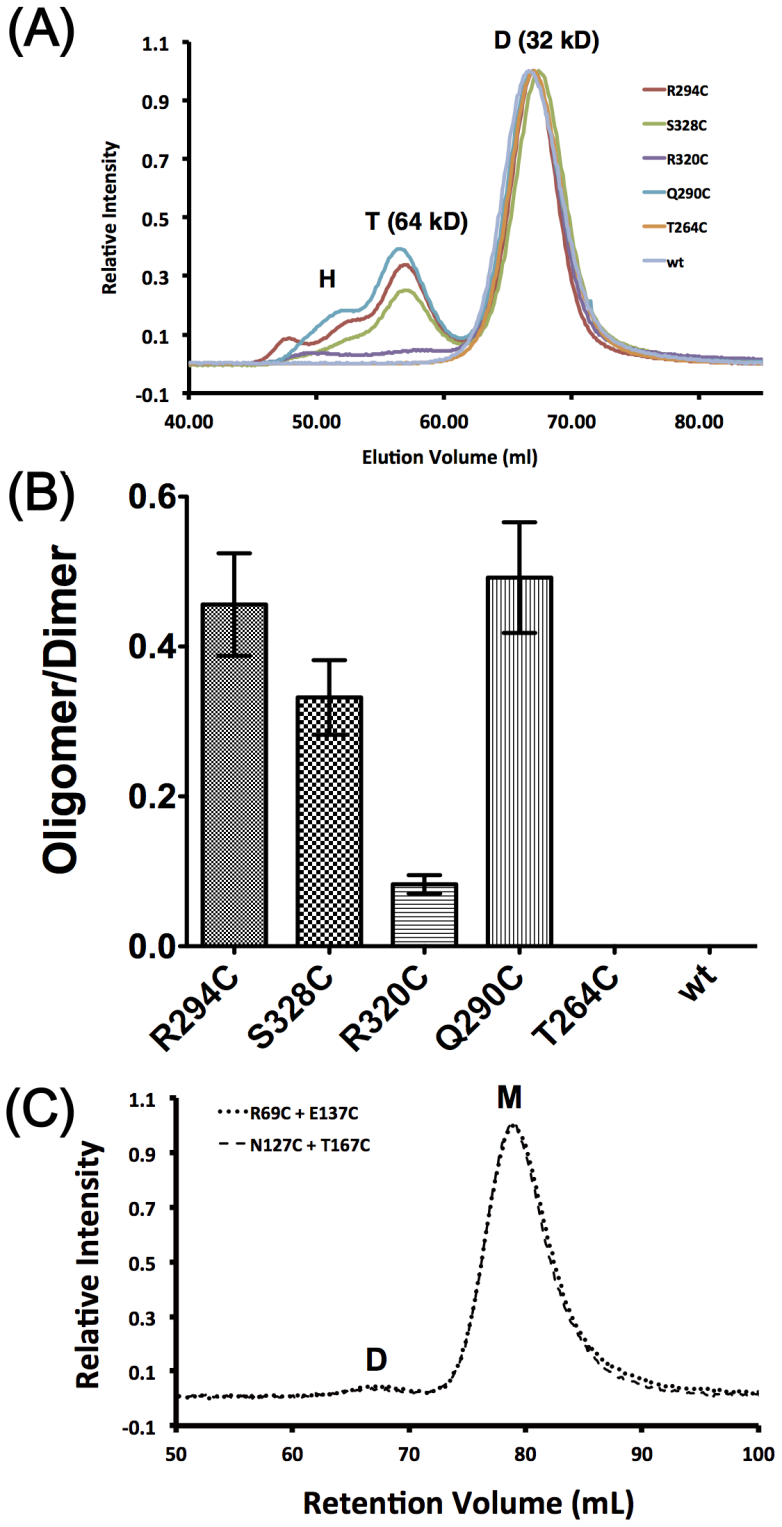
The CTD forms transient oligomers in solution. (A) Chromatograms of wild-type (wt) and mutant CTDs after disulfide trapping show that some CTD mutants are forming oligomers while others are not, as evidenced by the appearance of peaks H (for high-order oligomer) and T (tetramer). Relative intensities were normalized to the major peak representing dimeric CTD (peak D). (B) Bar representation of the oligomer/dimer ratios of the CTD mutants estimated by area integration of chromatogram profiles shown in (A). The amount of oligomer is the sum of tetramer and higher oligomers. Error bars represent fitting errors calculated from the residual between fitted and experimental elution profiles. Wild-type and the T264C mutant showed negligible oligomer formation. (C) SEC profiles of NTD mutants after disulfide trapping treatment. Each reaction contained 3 mg/ml of total protein with each mutant comprising half of the concentration. Reaction conditions were the same as those used for the CTD mutants. Only very little dimer (D) formation was observed for both NTD mutation pairs with most of the protein remaining in the monomer (M) form.

We then tested for the ability of these mutants to spontaneously form disulfide linkages at 150 mM salt concentration through size-exclusion chromatography. None of the mutants are located close enough to form intra-dimer disulfide linkages, thus any disulfide linkage must be due to inter-dimer protein disulfide bond formation. The elution profile from a Superdex-75/60 size-exclusion chromatography (SEC) column (molecular weight cut-off 75 kDa) of the CTD mutants, shown in Figure 2, fall in two groups: CTD_Q290C_, CTD_R294C_ and CTD_S328C_ had high oligomerization potential; whereas CTD_T264C_ and CTD_R320C_ had little to no oligomerization potential. We observed three major peaks with molecular weight of 32 kDa, 64 kDa and higher molecular weights for peaks D, T and H, respectively, in the chromatograms of CTD_Q290C_, CTD_R294C_ and CTD_S328C_. The first peak eluted out from the column, peak H, has a molecular weight outside of the resolution limit of the resin. These molecular weights correspond to dimer and tetramer for the D and T peaks. The populations of the oligomers (T + H) are plotted on Figure 2B. Our results are in general agreement with the proposed crystal packing structure of the CTD with Gln290, Arg294 and Ser328 having higher populations of oligomers. These results qualitatively suggest that the CTD of SARS-CoV N protein is capable of transient self-association through the oligomer interface identified in the crystal structure.

We also tested the oligomerization potential of the NTD since its crystal packing hinted at the possibility of NTD oligomerization [16]. We introduced Cys mutations at Arg69, Asn127, Glu137 and Thr167; sites that have the potential to form spontaneous disulfide linkages based on the trimeric crystal packing structure of the NTD [16]. According to the structure, Arg69-Glu137 and Asn127-Thr167 pairs are close in space and should have high probability of forming spontaneous disulfide linkages. However, we did not observe significant oligomer formation from our chromatograms (Figure 2C), suggesting that the NTD either does not form oligomers or forms oligomers through an unidentified intermolecular interface.

### Electrostatic screening drives self-association of SARS-CoV N protein CTD

**Figure 3:**
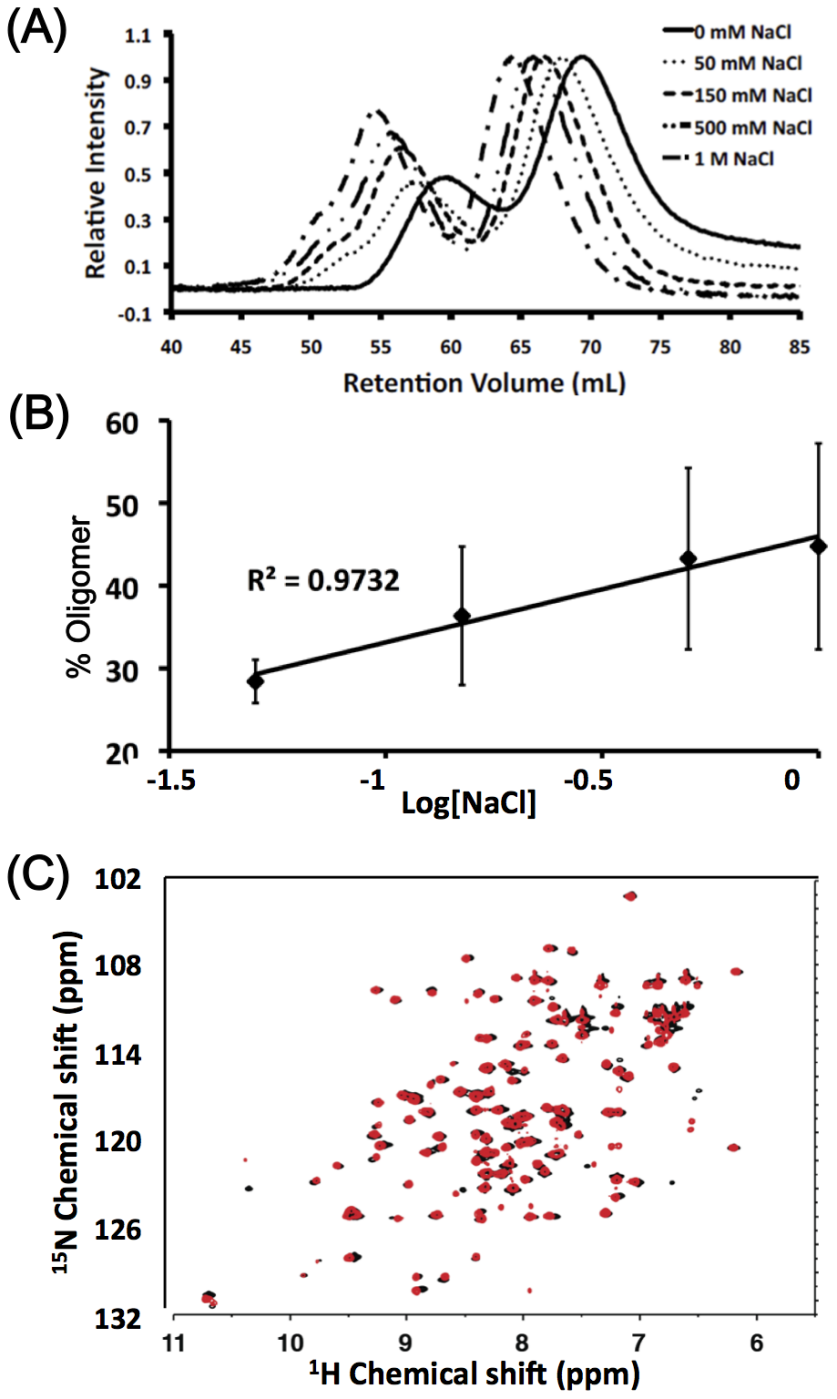
Increasing salt concentration leads to increased CTD_Q290C_ self-association. (A) Chromatogram of CTD_Q290C_ under different salt concentrations. The systematic shift is caused by nonspecific interaction of the protein with the Superdex column matrix in the absence of salt. (B) Oligomer percentage increases with increasing salt concentration in a semi-log manner. Data points (♦) and error bars represent the average and standard error of three independent experiments. (C) Overlay of ^15^N-edited HSQC spectra of CTD_Q290C_ in 50 mM NaCl (black) and 500 mM NaCl (red). Resonance patterns between the two are virtually identical, indicating that no structural changes occur at different salt concentrations.

SARS-CoV N protein is highly electropositive due to an abundance of basic residues. These charges are considered important for RNA binding, but they are also potentially deterring the self-association of the protein through electrostatic repulsion [11], [12]. The inter-protein charge-charge interaction can be affected by salt concentration, thus we first studied the effect of buffer salt concentration on trapped self-associated species of CTD_Q290C_ and the results are shown in Figure 3. The systematic shift of the chromatograms toward higher retention volume (lower molecular weight) with increasing salt concentration (Figure 3A) are due to non-specific adsorption of the protein to the Superdex matrix [24]. The relative amount of tetramer and larger oligomers in solution increases with increasing salt concentration (Figure 3B), suggesting that reducing charge repulsion by increasing salt concentration enhances self-association of CTD. Salt-induced conformational changes, electrostatic screening or a combination of both potentially could promote self-association of the protein [25]. However, one would expect the resonance positions of the ^15^N-edited HSQC of the CTD_Q290C_ in low-salt (50 mM NaCl) and high-salt (500 mM NaCl) conditions to be different if there were changes in the conformation. Since the two spectra are virtually identical (Figure 3C), one can rule out the possibility of conformational change-induced oligomerization.

### Phosphorylation-mimicking mutations increase self-association tendency of the DD_Q290C_

**Figure 4:**
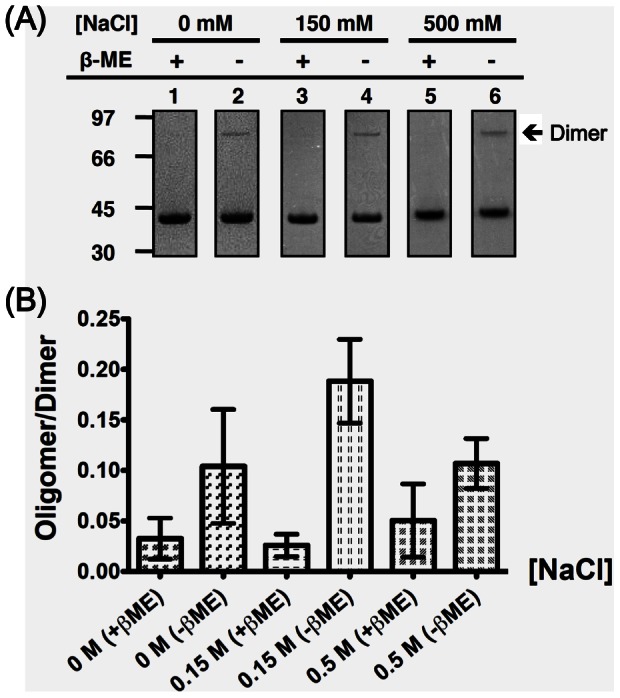
CTD acts as an oligomerization domain of DD. (A) Representative SDS-PAGE strips of DD_Q290C_ mutant after disulfide trapping treatment. Number on the left marks the molecular weight of the markers. The arrow denotes the position of disulfide-linked di-domain on the gel. Unlike CTD_Q290C_, DD_Q290C_ did not exhibit salt-dependent oligomerization as quantified in (B). The bars represent the average results from three independent experiments.

We further tested the oligomerization behavior of a Q290C mutant of the di-domain construct consisting of the NTD, the linker region and the CTD (DD_Q290C_, a.a. 45–365). We utilized a non-reducing denaturing polyacrylamide gel electrophoresis (PAGE) assay due to the large molecular weight of the DD dimer (∼ 70 kD), which is close to the void volume of the SEC column used for the CTD studies. Because the positions of the two Gln290 in the dimer are far apart, formation of disulfide bonds in the Q290C mutant would require at least two dimers to draw close together in space, such as formation of tetramers of higher order oligomers. Denaturation of the dimer under non-reducing conditions would form monomers, whereas under the same conditions, high-order oligomers would form a mixture of dimers (those that formed a disulfide bond) and monomers. So presence of dimer bands on a non-reducing denaturing gel would reflect the presence of tetramers or high-order oligomers. Consistent with our previous results, we also detected oligomers of the DD_Q290C_ as shown in Figures 4A and 4B. Interestingly, we saw little effect on the oligomerization of DD_Q290C_ when changing solution salt concentration. Although the reason for the discrepancy between DD_Q290C_ and CTD_Q290C_ is unknown, one possible explanation is that the additional positive charges of DD_Q290C_ require much stronger electrostatic screening (e.g. even higher salt concentrations) to have an observable effect on the oligomerization of the protein, akin to the electrostatic screening threshold observed in crystallization studies [26].

**Figure 5:**
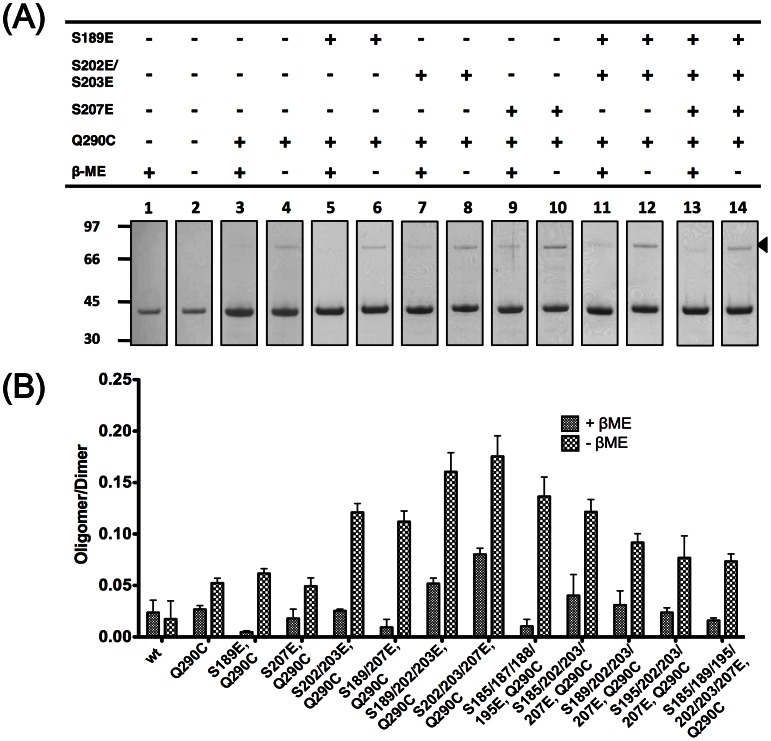
Effect of phosphomimicking negative charges on di-domain self-association. (A) Representative SDS-PAGE strips from disulfide trapping experiments of di-domain mutated at sites known to be phosphorylated by GSK3 and SRPK1. The arrow denotes the position of disulfide-linked di-domain on the gel. Strips from other mutants are included in the supplemental material. (B) Quantification of the self-association potential of all phosphomimicking mutants from this study. Bars represent average results from three independent experiments.

We further examined whether changing the electrostatic properties of the protein itself would affect transient self-association. We chose the putative phosphorylation sites on the flexible linker as our prime target, and assayed the effect of negative charges on N protein self-association by changing these sites from Ser to Glu in the DD_Q290C_ mutant [18], [19], [20]. In Figure 5, we observed that gradual introduction of negative charges on the unstructured linker had a positive effect on the oligomerization of the DD when compared to the DD_Q290C_ control, with maximum effect achieved when 3 negative charges were introduced per each chain (see Figure S1). Further increases in negative charges (S185/189/195/202/203/207E) were less effective in enhancing DD_Q290C_ oligomerization. We checked the overall structure of mutants mimicking putative phosphorylation with known kinases [19], [20], i.e. S189E, S207E, S202/203E and S189/202/203/207E with ^15^N-HSQC and found that the extra negative charges did not affect the overall structure of the DD (Data not shown), suggesting that electrostatic interactions rather than conformational changes are responsible for the increased tendency to self-associate. Overall, our results suggest that hyperphosphorylation of the LKR, which reduces the total positive charge of the N protein, can enhance and regulate oligomerization of DD through electrostatic effects.

## Discussion

Understanding the self-association of capsid proteins is a fundamental step towards unraveling the mechanism of viral self-assembly. The study of self-association of coronavirus N proteins poses a particular challenge due to the intricate arrangement of structured and disordered domains that have been implicated in protein self-interaction. The literature on how SARS-CoV N protein oligomerize is rather confusing and the mechanism by which N protein associate with RNA to form the helical RNP is also far from understood. It has been previously established that the SARS-CoV N proteins exist as dimers in solution with little indication of forming higher order oligomers in the absence of nucleic acids [8], [10], [14]. Through a combination of disulfide trapping and structure-based mutagenesis, we successfully trapped transiently interacting oligomers of the CTD, suggesting that transient self-association between dimers do occur at specific site(s). The Q290C mutation proved to be particularly useful in this study because it had little effect on the structure of the CTD and was electrostatically neutral, yet allowed for the selective detection of direct physical interaction between dimers. It enabled us to explore the effect of various variables (e.g. salt concentration and phosphorylation) on the oligomerization of the SARS-CoV N protein in a controlled environment. This study provides biochemical evidence that the CTD, but not NTD, is a transient intermolecular self-association site of SARS-CoV N protein. Our results further suggest that transient self-association can be regulated by lowering intermolecular electrostatic repulsion, either through environmental charge screening or charge modifications on the protein itself.

### Comparison with previous oligomerization studies of SARS-CoV N protein

Interestingly, our data is contrary to what was found in a number of previous studies. Luo et al. reported that oligomerization of a SARS-CoV N protein construct spanning residues 283–422 was not affected by salt [15]. Peng et al. found that SARS-CoV N protein phosphorylated by SRPK1 induced less oligomerization than its unphosphorylated counterpart [19]. The disagreement most likely lies in the differences between the cross-linking techniques employed by the different groups. Our experimental design requires that two dimers must physically interact with each other close to the mutation site before cross-linking can take place, whereas the use of a chemical cross-linker in the previous studies did not distinguish between dimers that arise from intra-dimer or inter-dimer associations. In Luo et al. 's construct, the loss of residues 248–282 not only strips away most of the positive charges on the surface of the dimer, but also results in the loss of three N-terminal helices, which could cause adverse effects on the structural integrity of the protein [12]. Over all, we believe that our method provides a more direct measure of the self-association of the basic building blocks involved in capsid assembly by taking into account the structural modularity of SARS-CoV N protein. It should also be noted that although the free-form N-terminal domain did not oligomerize in our system, under *in vivo* conditions it still could form oligomers in the presence of RNA as proposed by Fan *et al* for the N protein of the infectious bronchitis virus [5]. Other parts of the coronavirus N protein, e.g. so-called C-terminal tail, have also been implicated in protein oligomerization [15], [27]. In fact, the multitude of regions found to contribute towards oligomerization of coronavirus N proteins suggests that N protein self-association is an intricate affair involving inter-molecular interactions among multiple domains of the protein.

### Electrostatic repulsion as a means of modulating nucleocapsid assembly

Our results show that it is possible to change the oligomerization potential of SARS-CoV N protein by tinkering with the electrostatic characteristics of the system (Figures 3 and 4). Normally, the positively charged N protein exerts a repulsive force that prevents self-association. In the case of the CTD, we enhance the charge screening effect and reduce the intermolecular repulsion when increasing the salt concentration, thus resulting in enhanced oligomerization. However, this salt dependency was lost when we changed the system to the DD construct (Figure 4). This could arise from the large number of charges in the DD (+36 charges/dimer) *versus* in the CTD (+14 charges/dimer), which would make it more difficult to screen the charges using the salt concentrations in this current study. Interestingly, our observations could be related to those by Verheije *et al* [28]. They observed *in situ* that self-interaction of CTD was independent of RNA, whereas homotypic interaction of full-length N protein required the presence of RNA. One could speculate that the cellular environment provided enough charge screening for CTD self-association, whereas RNA was required to counterbalance the excessive charges of the full-length protein.

N proteins of coronaviruses are highly phosphorylated in infected cells [29], [30]. SARS-CoV has also been shown to be phosphorylated in cells by several kinases, mainly at the serine residues in the SR region [18], [19], [20]. However, the *in vivo* phosphorylation sites of SARS-CoV N protein in actual infection have not been mapped. Furthermore, Peng *et al.* reported that phosphorylation of SARS-CoV N at the SR-rich motif could modulate its translation inhibitory activity and also its multimerization activity [19], thus phosphorylation may play a role in regulating the viral life cycle. Our findings that electrostatic repulsion acts as a means of modulating nucleocapsid assembly and that an optimal number of charges is favored for multimer formation suggest a possible mechanistic roles for phosphorylation in the regulation of SARS-CoV nucleocapsid assembly. In a fully formed capsid, each N protein would be stabilized by a network of weak protein-protein interactions with adjacent N proteins and protein-RNA interactions with the viral genome. The protein-RNA interactions neutralize the excessive charges on the N protein, thus negating the effect of electrostatic repulsion. Under these conditions, extensive phosphorylation of SARS-CoV N protein is not necessary and the protein can maintain a hypophosphorylated state within the virion as proposed in a past study [20]. This hypophosphorylated state does not necessarily imply complete dephosphorylation. In fact, it would be advantageous to “tune” the N protein such that it retained a small number of phosphorylated sites even in the assembled form (e.g. the “di-phosphorylated” mimics in Fig. 5B). One could then envision that further dephosphorylation events would enhance uncoating of the viral RNA due to electrostatic repulsion between N protein dimers (e.g. “mono-phosphorylated” mimics in Fig. 5B). Such a possibility has been proposed for JHMV, a neurotropic strain of mouse hepatitis coronavirus (MHV), where dephosphorylation of the N protein by an endosomal-associated cellular protein phosphatase was found to facilitate viral infections [31]. Newly produced N protein, on the other hand, would be able to bind to RNA with high affinity, but capsid formation could be impaired due to lack of protein-protein interactions caused by residual electrostatic repulsion. In this case, hyperphosphorylation of N protein could become a critical assisting factor, i.e. a “last straw” in neutralizing excessive electrostatic forces, in nucleocapsid assembly (e.g. “tri-phosphorylated” mimics in Fig. 5B).

Electrostatic repulsions have been shown to play similar roles in other viruses, where N proteins often have excessive positive charges. Hepatitis B virus (HBV) N protein, for example, has an isoelectric point of 9.93, and forms icosahedral capsids rather than helical ones found in coronaviruses. An increase in the solution salt concentration has been shown to induce the formation of empty HBV capsids, and theoretical studies have attributed the phenomenon to charge screening effects [32], [33]. Recently, a “charge balance hypothesis” was proposed to explain hepatitis B virus (HBV) capsid stability, assembly, RNA encapsidation, and DNA replication [34], [35], [36]. This hypothesis emphasized the importance of a balanced electrostatic interaction between the positive charge from the arginine-rich domain (ARD) of the core protein (HBc) and the negative charge from the encapsidated nucleic acid. Our results reinforce the common theme that electrostatic repulsion is a universal mechanism of capsid assembly modulation in viruses.

### Coupled nucleic acid binding and self-association

The observation of trapped transient oligomer shown in the present study does not mean that CTD acts as the nucleation center for the capsid formation. In fact, we had previously shown that N protein interacts with nucleic acid at multiple sites and with high affinity, thus N-RNA interaction presumed to be the driving force for the ribonucleoprotein (RNP) formation [13]. In this RNA binding-coupled RNP packing scenario, the multitude of weak protein-protein interactions reinforces the protein-RNA interaction and contributes towards the assembly of a stable RNP, which guarantees the formation of nucleocapsids containing genetic material. The modular structure and multiple sites of moderate RNA binding affinity of the N protein not only allow the packaging of a stable RNP but also offer an energetically favorable condition for the expression of the viral genomic information. One can envision an unzipping mechanism for unwinding of the viral RNA molecule and dissociation of the RNA molecule from the N protein in a stepwise manner, one module at a time, without the need to overcome a high-energy barrier. This is consistent with the concept for virus assembly that capsid proteins associate through locally weak interactions to form globally stable structures [25]. Weak interactions between capsid proteins minimize formation of kinetic traps, allow a greater degree of regulation of assembly, and may be essential for viruses where dissociation is part of the virus life cycle. On the other hand, newly synthesized N protein would also be involved in many RNA-protein or protein-protein interactions, most likely modulated by N protein phosphorylation state, affecting viral and host processes other than nucleocapsid assembly [2]. More works need to be performed to understand the effect of N protein phosphorylation on viral and host processes other than nucleocapsid assembly.

## Conclusion

By disulfide trapping technique we measured the amount of transient oligomers of N protein mutants with strategically located cysteine residues and showed that SARS-CoV N protein is capable of transient oligomerization in solution through the CTD in the absence of nucleic acids. The data is compatible with the helical oligomer packing model of N protein observed in crystal. This transient oligomerization process is driven by the neutralization of excessive charges on the protein, which can be accomplished through changes in environmental salt concentration and protein phosphorylation. The data is consistent with a general mechanism whereby the electrostatic repulsion between N protein molecules acts as an oligomerization switch. It also reinforces the concept of RNA-binding coupled RNP packaging in SARS-CoV.

## Supporting Information

Figure S1 **Representative strips from SDS-PAGE of selected DD mutants after disulfide trapping.** The arrow denotes the trapped species originating from tetramers or higher order oligomers.
(PDF)
