# Supplementary material for: Transient Oligomerization of the SARS-CoV N Protein – Implication for Virus Ribonucleoprotein Packaging
Source: PLoS One. 2013 May 23;8(5):e65045. doi: 10.1371/journal.pone.0065045 (PMC3662775; doi:10.1371/journal.pone.0065045)
Supplement: Figure S1 — Representative strips from SDS-PAGE of selected DD mutants after disulfide trapping. The arrow denotes the trapped species originating from tetramers or higher order oligomers. (PDF) [file pone.0065045.s001.pdf]

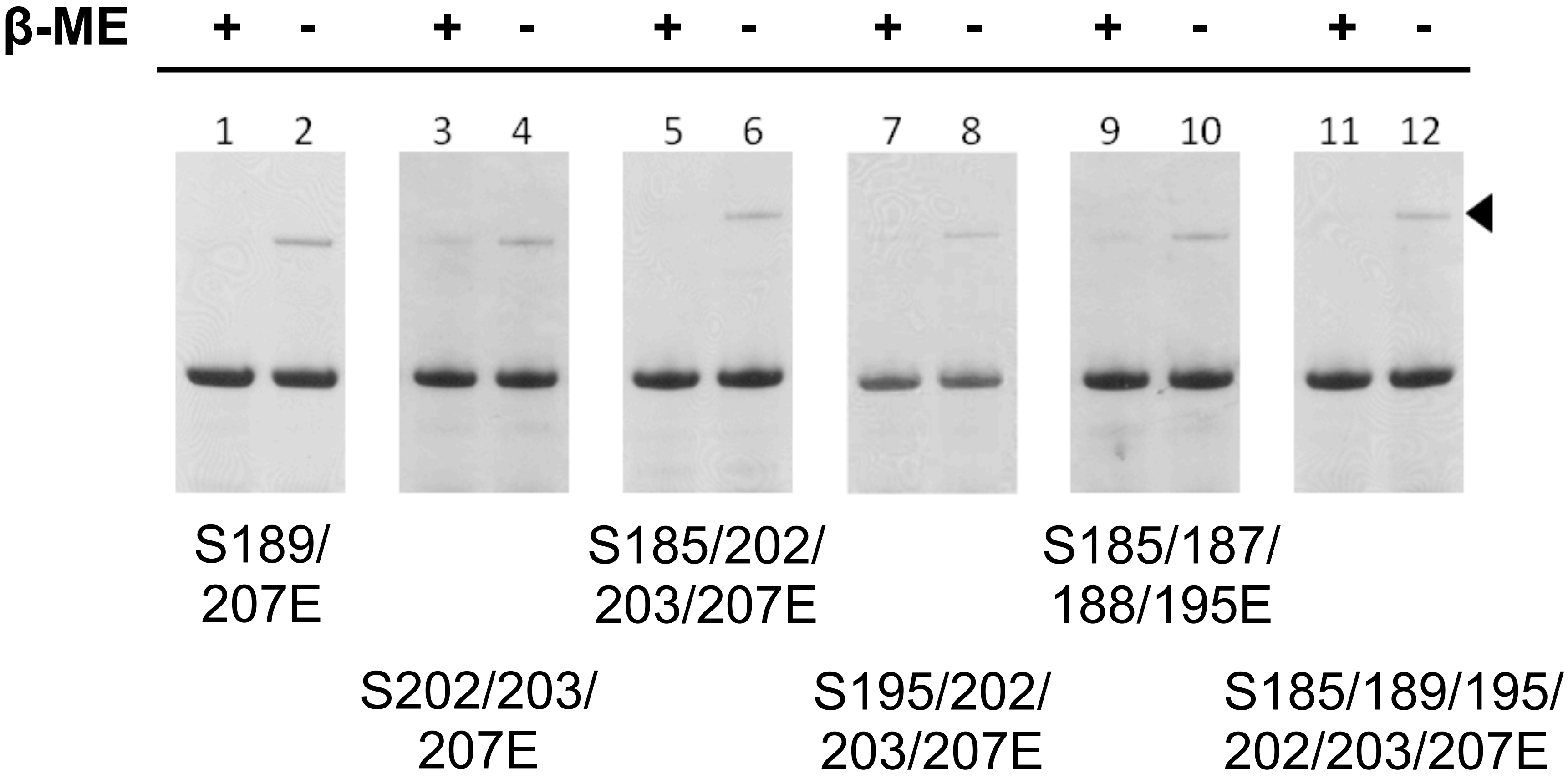

Figure S1. Representative strips from SDS-PAGE of selected DD mutants after disulfide trapping. The arrow denotes the trapped species originating from tetramers or higher order oligomers.
